# Position Detection System for Moving-Magnet Linear Motors Based on a Magnetoresistive Sensor Array

**DOI:** 10.3390/s25041019

**Published:** 2025-02-08

**Authors:** Jun Wang, Xiang Chen, Quyan Chen, Qing Xi, Haiyang Sun

**Affiliations:** 1 College of Information Science and Technology, Nanjing Forestry University, Nanjing 210037, China; wangjun@njfu.edu.cn (J.W.); cxiang@njfu.edu.cn (X.C.);; 2 Jiangsu Oubang Motor Manufacturing Co., Ltd., Dongtai 224200, China; 3 Wuxi Hongyi Intelligent Technology Co., Ltd., Wuxi 214000, China; xiqing722@yeah.net

**Keywords:** magnetoresistive sensor array, position detection, moving magnet, linear motor

## Abstract

The moving-magnet linear motor has received considerable attention in the development of logistic and factory automation in recent years. A reliable position detection system is the key to achieving the precise position and control of the motor. At present, the magnetic grid-scale and grating-scale are the most widely used traditional detection methods. However, these are not suitable for position detection with moving-magnet linear motors. They have the disadvantages of being easy to disturb, having a high cost, and exhibiting a limited measurement range. In this work, a moving-magnet linear motor position detection system based on an array of magnetoresistive sensors is used. The array is configured by arranging the magnetoresistive sensors at equal intervals along a line parallel to the trajectory of the armature. Then, the permanent magnet is fixed on the rotor and detected by sensors. When the rotor crosses the sensors in a parallel line, the changes in the magnetic field cause the magnetoresistive sensors to output two voltage signals directly proportional to the corresponding position changes. The signals are collected by the AD7606 and transmitted to the FPGA and STM32 controller for data processing, and the actual position of the rotor is calculated. This method has no length limitation and can be used for long-distance position detection. The experimental results show that the position detection system has a higher linear correlation coefficient compared with the magnetic grid ruler, in addition to a capability of ±9 μm accuracy, which verifies the validity of the position detection method for the moving-magnet linear motor.

## 1. Introduction

Traditional linear transmission systems often use rotary motors as their power source to convert rotary motion into linear motion through a transmission device [1,2]. Because of the installation of additional mechanical components, there are problems such as energy loss, noise, mechanical wear, and slow response time. Consequently, the system’s speed and acceleration make it difficult to meet the demands of some high-precision and high-response speed applications, such as those involving industrial robots and multi-axis machining centers; therefore, linear motors have been introduced. This paper studies the displacement detection of linear motors. To overcome the above problems, some experts use linear motors as a power source [3,4]. As a new type of motor structure [5,6,7], a permanent-magnet synchronous linear motor has the advantages of compact structure, light weight, high precision, and high efficiency in linear transmission. It can overcome the limitations of traditional rotary motors and gradually become the primary choice for many linear transportation applications. At present, permanent-magnet synchronous linear motors are divided into two types, including the moving-coil type and the moving-magnet type [8,9,10]. The moving-coil linear motor’s armature consists of a coil winding used as a rotor. The coil needs to be connected to the power supply when the rotor is in linear motion. The length of the power cable significantly limits the extent of the motor’s linear displacement. The motor can only move within a short linear distance, and is not capable of use across a long linear distance. The moving-magnet linear motor’s armature is composed of permanent magnets collectively utilized as a rotor or mover, and its stator consists of a sequential arrangement of many coil windings. Therefore, the rotor, in the course of its linear movement, will not be disturbed by the cable. The straight-motion length will not be limited, as long as the stator coil windings are laid along a sufficiently long path. It can achieve precise long-range servo control. With more and more applications of the moving-magnet linear motor becoming apparent, the position detection method relative to the armature has attracted more attention [11,12]. In the existing research relating to position detection for a moving-magnet linear motor’s armature [13,14], the linear-sensor methods of magnetic grids and optical gratings are the ones most commonly used [15,16,17,18]. Magnetic grids are low-cost and easy to install in most industrial applications. However, the position decoders of magnetic grids need to be installed on the rotor, and the detection distance is limited due to the electric wires connected to the power source. Additionally, the magnetic scales need to be replaced at regular intervals because of demagnetization problems [19,20,21]. Optical gratings can achieve high measurement accuracy, but have high demands as to the conditions of the operating environment. Oil and dust affect the performance of the grating and cause measurement errors. The mechanism needs to be sealed for use in order to avoid the influence of the environment. Therefore, optical gratings are also not suitable for moving-magnet linear motor applications. In order to realize a low-cost and stable position-detection method, magnetoresistive sensors have been considered for use [22].

### 1.1. Magnetoresistance

In 1856, Kelvin [23] discovered the phenomenon of magnetoresistance (MR) in ferromagnetic metals. Magnetoresistance is defined as the change in the electrical resistance of a material when an external magnetic field is applied. Since then, there have been growing numbers of experts and research topics focused on the magnetoresistance effect and materials and structures exhibiting magnetoresistance-based properties. Currently, the most well-established types include anisotropic magnetoresistance (AMR), giant magnetoresistance (GMR), and tunnel magnetoresistance (TMR). These effects have led to the mature development of products for both angle detection and position detection. These sensors are widely used in automotive applications, consumer electronics, and industrial equipment.

#### 1.1.1. Anisotropic Magnetoresistance (AMR)

**Figure 1:**
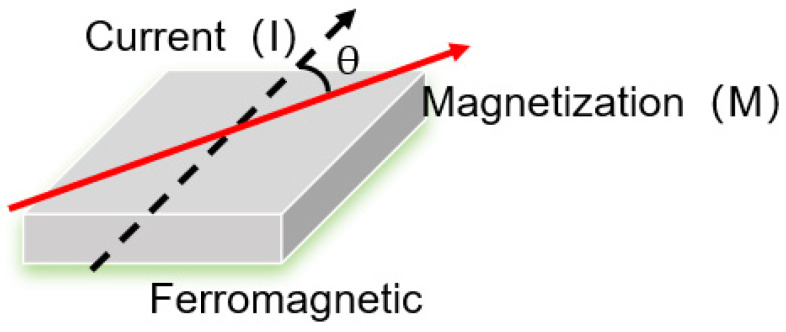
Schematic diagram of anisotropic magnetoresistance (AMR).

The change in resistance (∆R) depends on the angle (θ) between the current and the magnetization direction of the metal [24], primarily manifested in the influence of the rotating magnetization state in ferromagnetic materials on the direction of the current. In ferromagnetic metals, when the magnetization direction is parallel to the current direction, the resistance reaches its maximum value. When the magnetization direction is perpendicular to the current direction, the resistance reaches its minimum value (Figure 1). The magnitude of the anisotropic magnetoresistance (AMR) in ferromagnetic metals can be calculated using Equation (1). Currently, the AMR effect has been widely applied in miniaturized and high-sensitivity magnetic field sensors. Researchers are exploring materials and structures with even higher sensitivity.

(1) $$AMR=\frac{\rho_{\left|\right|}-\rho_{⊥}}{\rho_{⊥}}$$

where $\rho_{\left|\right|}$ is the resistivity when the current is parallel to the magnetization, and $\rho_{⊥}$ is the resistivity when the current is perpendicular to the magnetization.

#### 1.1.2. Giant Magnetoresistance (GMR)

**Figure 2:**
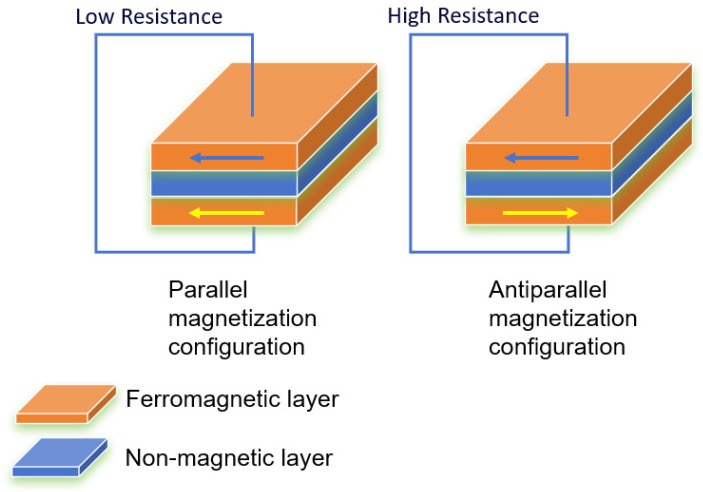
Schematic diagram of giant magnetoresistance (GMR).

In 1988, Fert and Grünberg independently discovered the giant magnetoresistance (GMR) effect in different laboratories [25]. Their research demonstrated that the change in resistance in thin films composed of alternating layers of ferromagnetic materials and non-magnetic metals (such as copper) can be quite significant, reaching up to several tens of times. This effect is primarily due to the scattering of spin-polarized electrons between different magnetic layers. The high resistance values can be observed when the magnetization of two ferromagnetic layers is anti-parallel, and the low resistance state can be observed when the magnetizations are at a parallel state (Figure 2).

#### 1.1.3. Tunneling Magnetoresistance (TMR)

**Figure 3:**
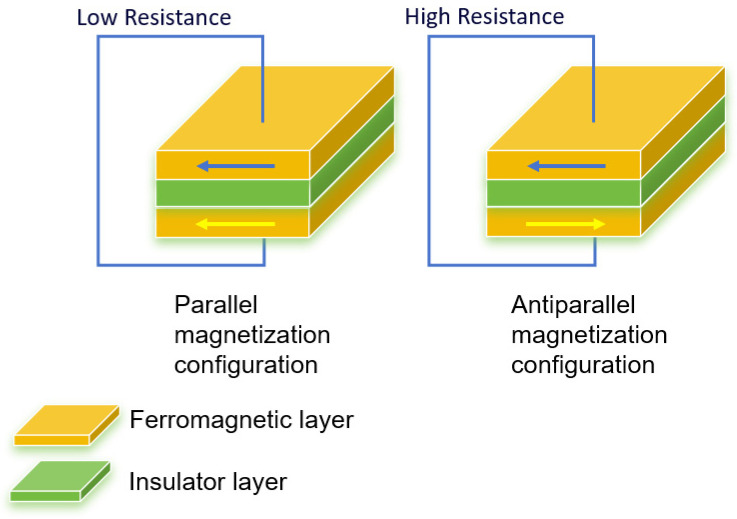
Schematic diagram of tunneling magnetoresistance (TMR).

In the 1970s, researchers began to explore the tunneling phenomenon associated with electrons in magnetic materials. Miyazaki and Tezuka were the first to observe that the resistance in a magnetic tunnel junction changes in response to an external magnetic field, a phenomenon that later became known as tunnel magnetoresistance (TMR) [26]. TMR uses a magnetic multilayer configuration similar to that of the GMR. The basic unit of the TMR multilayer system is the magnetic tunnel junction (MTJ), which follows the structure of ferromagnetic metal/insulator/ferromagnetic metal. The insulator layer is applied instead of the non-magnetic layer of the GMR multilayer system (Figure 3) [27].

The formula for calculating the magnetic resistance of the tunnel junction is as follows:

(2) $$\frac{\Delta G}{G}=\frac{G_{T}-G_{B}}{G_{T}}=\frac{{2PP}^{\prime }}{{1+PP}^{\prime }}$$

In the equation, $G_{T}$ and $G_{B}$ denote the conductance values when the magnetization directions of the two layers of ferromagnetic metal are in the parallel and non-parallel states, respectively, and P and $P^{\prime }$ denote the self-polarization rates associated with the effective transport state density of the two layers of ferromagnetic metal, respectively.

### 1.2. Giant Magnetoimpedance

Regarding the discovery of the giant magnetoimpedance (GMI) effect, it is widely recognized in the academic community that two research groups independently and nearly simultaneously identified this phenomenon. A publication by R. S. Beach and A. E. Berkowitz, along with a publication by L. V. Panina and K. Mehri, both reported the discovery of the GMI effect in amorphous wires [28,29]. These researchers made a quantitative analysis of the phenomenon which is rooted in classical electrodynamics. They showed the characteristic magnetic response of the wire impedance from the experimental and theoretical field dependencies of the MI effect.

The GMI effect, which refers to the significant changes in the impedance of an amorphous wire under the excitation of alternating current, occurs as the external magnetic field applied along the wire’s axis varies. The change rate of the impedance can reach up to 50% under a magnetic field of a few Oersted (Oe), which is an order of magnitude larger than the giant magnetoresistance (GMR) observed in metallic multilayer films such as Fe/Cu or Co/Ag at low temperatures and high magnetic-field strengths.

The most commonly used expression for the GMI effect is the magnetoimpedance ratio, ΔZ/Z, defined as

(3) $$\frac{\Delta Z}{Z}=\left[Z\left(H\right)-Z\left(H_{max}\right)\right]/Z\left(H_{max}\right)$$

where $H_{max}$ is the maximum axial DC magnetic field (usually up to a few kA/m).

The performance of the GMI effect is highly dependent on the material’s magnetic anisotropy, soft magnetic properties, skin effect, and microstructure. Therefore, material research is the core driving force behind the advancement of GMI technology. Many experts have studied thin film materials for MI sensor applications. González, J.M. et al. [30] employed broadband ferromagnetic resonance (FMR) measurement techniques to assess the performance and manufacturing consistency of multilayer thin-film structures for magnetoimpedance sensors. This work provides an efficient evaluation tool for quality control in magnetoimpedance sensors and analyzes the microwave resonant absorption of magnetron-sputtered Permalloy-based magnetic films suitable for magnetoimpedance sensor applications. Corrêa, M.A. et al. [31] explored the influence of geometric structures on the magnetoimpedance effect by designing NiFe/Ag multilayered (ML) films and ML/SiO_2_/Ag/SiO_2_/ML structured multilayered (SD) films produced by magnetron sputtering. Aiming to optimize the magnetoimpedance (MI) performance, they compared two types of multilayered films for the different MI behaviors and explained the MI changes of sample geometry and the mechanisms observed in distinct frequency ranges. These features may be useful for technological applications of frequency-tunable MI-based magnetic-field sensors.

These two studies enhance the comprehension of the thin-film magnetoimpedance effect from the perspectives of process stability and structural design, jointly driving the practical development of high-frequency magnetic sensor technology.

### 1.3. Introduction to the Research Work

In this study, a moving-magnet linear motor position detection system based on an array of magnetoresistive sensors is designed. The study aims to explore the performance of magnetoresistive sensor arrays in position detection systems for moving-magnet linear motors, and to select the most suitable magnetoresistive sensors for use in displacement detection systems for moving-magnet linear motors through comparative analysis. This system not only detects the absolute position of the mover in real time with high accuracy, but also, the sensor array can be modularly spliced for detection. Additionally, the detection length can be increased at any time and any place, making it suitable for the long-distance motion detection of the mover. This study aims to develop a displacement detection system for moving-magnet linear motors. The mover of the moving-magnet linear motor consists of a permanent magnet and the stator consists of coils. Therefore, the mover does not require a power supply or cable carrier, and has the advantages of high precision, fast speed, long displacement, and high motion-control efficiency. By measuring the exact position of the mover, the stator coil voltage at the corresponding position can be controlled to control the starting, stopping, or speed of the mover. Multiple movers can be installed in one linear motor, and separate control of multiple movers can be realized, which greatly enhances the working efficiency of the actual production line. The main contributions of this paper are as follows.

1. According to the sensor principle and the operating mode of the linear motor’s armature, the arrangement of sensors and the spatial relative position of the permanent magnets and sensors for detection are designed.
2. The output signals from each sensor may not be completely consistent, resulting in fluctuations of output voltage. By digitally processing the collected signals in the FPGA, errors can be eliminated, ensuring that the average of the two sine signals output by each sensor is the same, improving the displacement detection accuracy.
3. Displacement calculations are performed in the STM32, and the real-time position of the actuator is output to the host computer for display.
4. The final system is tested to verify the effectiveness of this method.

## 2. Composition and Detection Principle

### 2.1. Introduction to the Detection System

**Figure 4:**
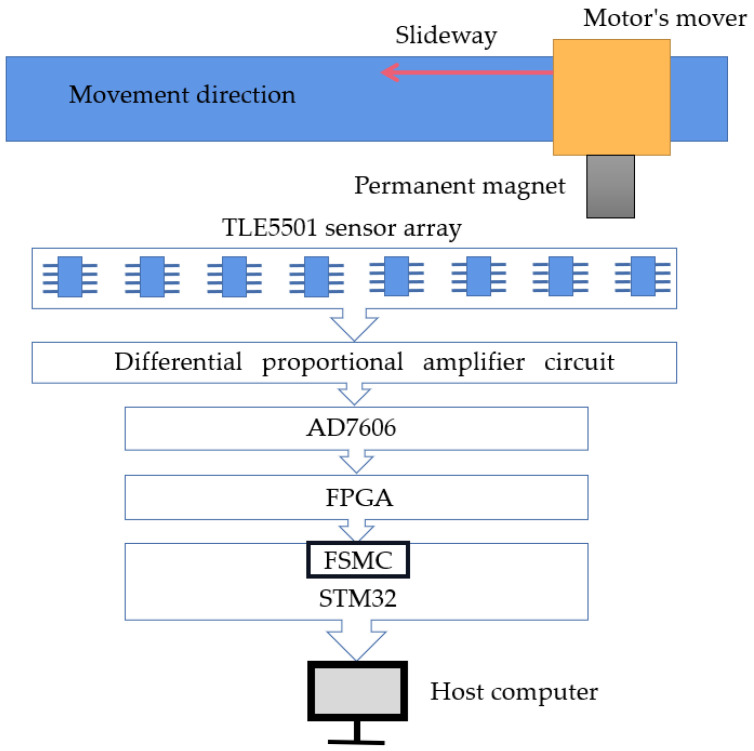
Overall block diagram of the position detection system program.

This article describes a position detection method based on the TLE5501 (Infineon Technologies AG, Munich, Germany) magnetoresistive sensor array. The magnetoresistive sensors are arranged at equal intervals along a straight line, and the detection range of each sensor is sequentially “spliced” in the form of an array, arranged in parallel next to the guide rail on which the mover runs. The motion of the mover causes the permanent magnet to pass through the sensor, and the sensor outputs two sinusoidal voltage signals, achieving long-distance linear position detection. Figure 4 is the overall block diagram of the system scheme, including the TLE5501 sensor array, a differential proportional amplification circuit, an AD7606 analog-to-digital conversion module (Shenzhen Convince Technology Co., Ltd., Shenzhen, China), an FPGA and STM32 module (Nexperia Co., Ltd., Shenzhen, China), and the PC host computer.

### 2.2. Equivalent Magnetic Charge Model (EMC)

The equivalent magnetic charge model assumes that there is a body charge of density $\rho_{m}$ inside the magnet and a surface charge of density $\rho_{\mathrm{ms}}$ on the surface of the magnet.

According to the equivalent magnetic charge model, the body charge density $\rho_{m}$ present in the permanent magnet is as follows:

(4) $$\rho_{m}=-\mu_{0}\nabla \cdot M$$

In the equation, $\mu_{0}$ is the vacuum permeability, and *M* is the magnetization strength.

Inside a uniformly magnetized magnet, there is M = 0, and hence the body charge density $\rho_{m}=0$. However, at the boundaries there is still a surface charge $\rho_{ms}$ on the magnet boundaries due to the discontinuity of M:

(5) $$\rho_{ms}=-\mu_{0}n\cdot M$$

In the equation, n is the outer normal unit vector of the magnet boundary.

According to Gauss’s law in Maxwell’s system of equations, the scalar magnetic potential at an arbitrary point in space generated by a permanent magnet can be obtained as

(6) $$\varphi_{m}=\frac{1}{4\pi \mu_{0}}{\int \int \int }_{\Omega_{1}}\frac{\rho_{m}}{R}dv+\frac{1}{4\pi \mu_{0}}{\int \int \int }_{\Gamma_{2}}\frac{\rho_{ms}}{R}ds$$

Applying the scattering theorem, this can be rewritten as

(7) $$\varphi_{m}=\frac{1}{4\pi }{\int \int \int }_{\Omega_{1}}M\cdot \nabla \frac{1}{R}dv$$

According to the relationship between the scalar magnetic potential *φ* and the magnetic-field strength *Hm*, *Hm* = $\varphi_{m}$; the magnetic-field strength in the space around the permanent magnet can be determined:

(8) $$H_{m}=\frac{1}{4\pi \mu_{0}}\left({\int \int \int }_{\Omega_{1}}\frac{\rho_{m}R}{R^{3}}dv+{\int \int \int }_{\Gamma_{2}}\frac{\rho_{ms}R}{R^{3}}ds\right)$$

In the equation, R is the vectorial diameter from the source point (inside the permanent magnet) to the field point (the solution point). R is the distance from the source point to the field point, $\Omega_{1}$ is the permanent magnet integration region, and $\Gamma_{2}$ is the boundary of the permanent magnet.

### 2.3. Comparison and Introduction of Magnetic Sensors

Magnetic sensors have been used for measuring the magnitude, the direction, and the relative change of the magnetic field. There are two main categories of magnetic-field sensors: the magnetoimpedance sensor and the magnetoresistive sensor. The magnetoimpedance sensor utilizes the phenomenon known as the GMI effect, which is the large response of electrical impedance in certain materials caused by an external magnetic field. This causes an increase in the impedance of the wire with increasing frequency. The GMI sensor and integrated circuit were developed by the Aichi Steel Co., Tokai, Japan, in October 2001. Over the last two decades, many subsequent applications and studies have been developed. On the other hand, magnetoresistive sensors have been developed over time based on the magnetoresistive [32,33] effect, and many different types of magnetoresistive sensors have now been developed, including anisotropic magnetoresistance (AMR) [34], giant magnetoresistance (GMR), and the latest, tunneling magnetoresistance (TMR). They are summarized in the following:

1. Sensitivity and magnetic-field range: TMR sensors are extremely sensitive and accurate, capable of accurately measuring changes in weak magnetic fields. With an MR (magnetoresistive) ratio of up to 100%, the output signal sensitivity is significantly improved compared to AMR and GMR sensors, reaching improvements of 13.3 times and 3.5 times, respectively. This feature establishes TMR sensors as the preferred choice for applications that require high sensitivity and an extensive measurement range of the magnetic field. GMI sensors also exhibit a wide sensitivity range, extending from the millimeter-tesla region to the weak magnetic field of the pico-tesla.
2. Energy consumption: TMR sensors have low power requirements. Therefore, TMR is a very important approach for devices that require long-term operation or battery power, and especially for sensor arrays. Their low power consumption helps to extend the life of the device and reduce energy costs. AMR and GMR have relatively higher levels of power consumption due to the fact that their supply currents are larger. GMI sensors exhibit low levels of power consumption due to the pulse interval independence associated with the GMI effect.
3. Working temperature: TMR has good linearity and stability and shows a good linear relationship between the output signal and the input magnetic field. It also has a wide operating temperature range and can work stably under various environmental conditions. This ensures the stability and reliability of TMR sensors in harsh environments. At the same time, temperature stability also affects the performance and energy consumption of the sensor, so the temperature stability of TMR is very beneficial for power management. GMI sensors have a limited working temperature range due to the difficulty in integrating the sensing wire and the GMI effect being affected by temperature in practical applications.
4. Cost: The prices of AMR, TMR, and GMI sensor chips are similar, while GMR sensor chips are relatively expensive.

The comparisons of magnetic sensors are listed in Table 1.

Based on the above considerations, this study has chosen TMR sensors, which are associated with small size, high stability, low power consumption, and high sensitivity. The following is a description of the TMR sensor.

**Figure 5:**
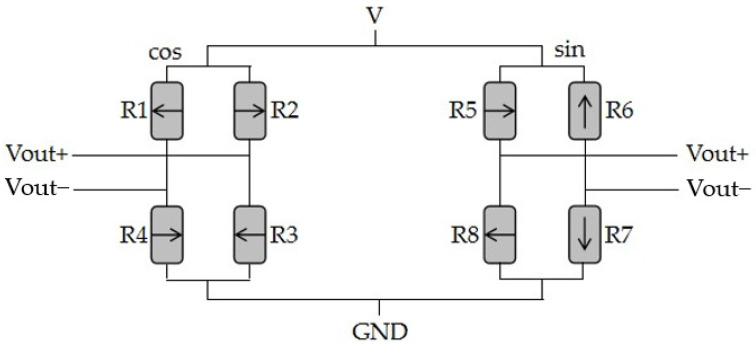
A single sensor, the equivalent of two Wheatstone bridges.

The TLE5501 consists of eight TMR devices. The sensor is made by micromanipulation of magnetoresistive film stack structures. This includes preparing a desired circuit pattern using processes such as photolithography, vapor deposition, and stripping, and then vapor deposition of electrode materials such as gold and copper onto the magnetoresistive film stack structure to form the desired circuit connections. The structure can be described as being equivalent to two Wheatstone bridges [35]. It is shown in Figure 5.

From this, we can see the following:

(9) $$V_{out\left(cos\right)}=V\mathrm{Scos}\theta$$

(10) $$V_{out\left(sin\right)}=V\mathrm{Ssin}\theta$$

**Figure 6:**
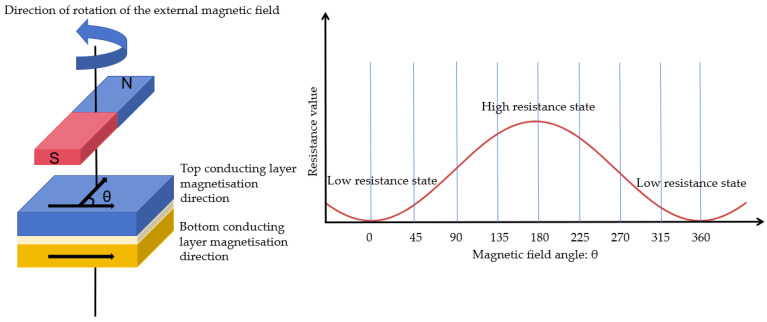
Schematic diagram of clamping resistor.

In the formula, V is the supply voltage of the bridge, and S is the material constant. The θ is the angle between the magnetization direction of the top conducting layer and bottom conducting layer, where the magnetization direction of top conducting layer follows the direction of external magnetic field. So θ is the angle between the external magnetic field and the magnetization direction of the bottom conducting layer. This is shown in Figure 6.

### 2.4. Position of the Sensor Relative to the Permanent Magnet

**Figure 7:**
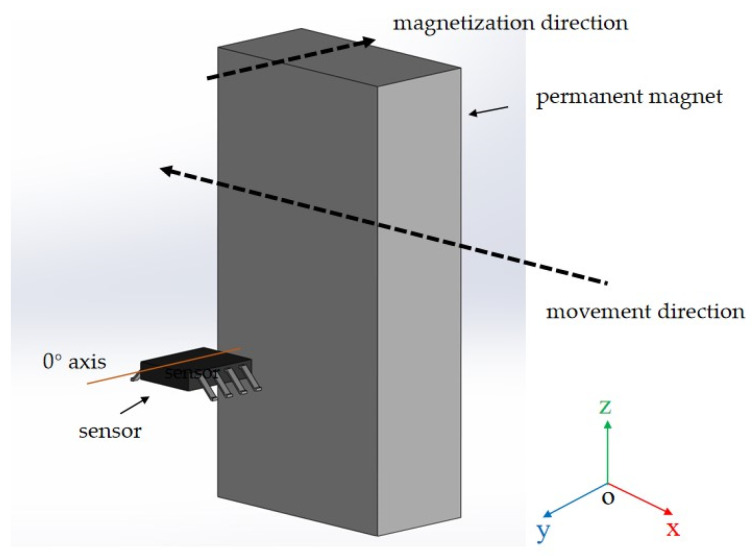
Schematic diagram of the spatial position of the sensor relative to the permanent magnet in case 1.

Since the magnetoresistive sensor is only sensitive to magnetic fields parallel to the sensor surface, magnetic fields perpendicular to the sensor surface have little effect on the sensor’s output. The permanent magnet used in this article is a thickness-direction-magnetized permanent magnet. There are two cases regarding the spatial relationship between the sensor and the permanent magnet. The first case is illustrated in Figure 7.

**Figure 8:**
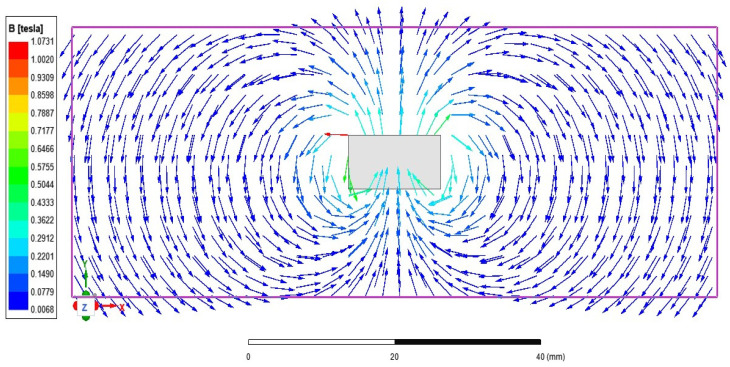
Simulation of magnetic induction vector in the XY plane.

Case 1:

At this time, the magnetization direction of the permanent magnet is parallel to the *y*-axis. Since the magnetoresistive sensor is only sensitive to magnetic fields parallel to the sensor surface (XY plane), the only need to be focused on is the magnetic field of the permanent magnet in the XY plane. Figure 8 shows the Maxwell simulation of the magnetic induction intensity vector in the XY plane corresponding to case 1.

**Figure 9:**
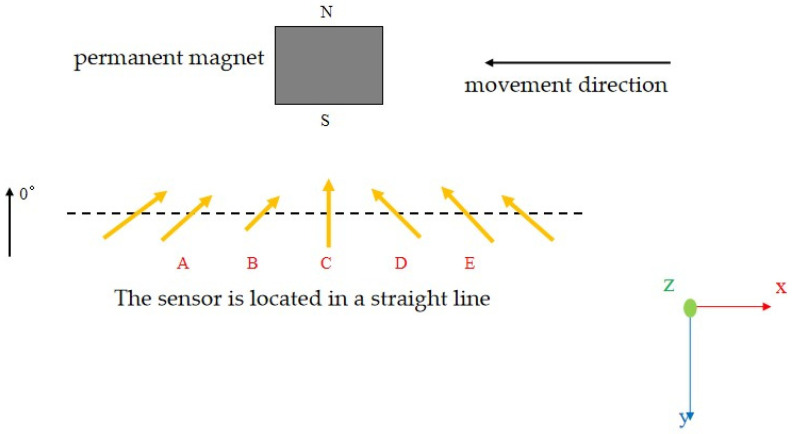
Schematic of the magnetic induction vector of the line where the XY plane sensor is located.

Figure 9 shows the magnetic induction vector on the line where the sensor is located in the XY plane. As can be seen in Figure 7, the line at position C of the magnetic induction vector coincides with the 0° direction of the sensor. The line with the magnetic induction vector at position B makes an angle of 45° with the 0° direction of the sensor. The line at position D of the magnetic induction vector makes an angle of −45° with the 0° direction of the sensor. The further towards the sides one is, the closer the angle is to the +90° and −90° angles.

**Figure 10:**
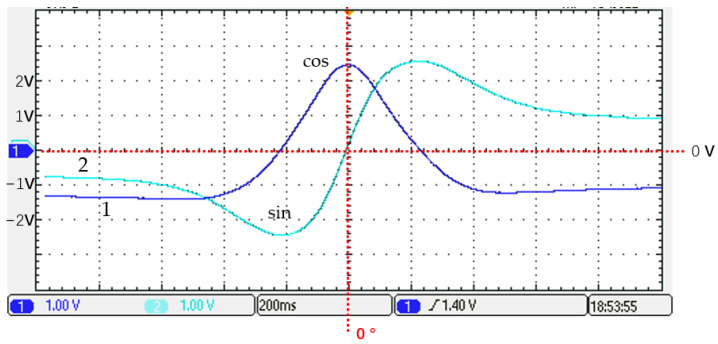
The magnetoresistive sensor output waveform in case 1.

Figure 10 shows the output waveform of the magnetoresistive sensor when the permanent magnet moves parallel to the magnetoresistive sensor along a straight line at a constant speed in case 1. The output waveform of the magnetoresistive sensor is shown in Figure 10. Obviously, when the permanent magnet crosses from one side of the sensor, the angle ranges from −90° to +90°. The cos waveform in this figure is obviously negative, indicating that the angle in this case has a significant deviation, and the angle read by the sensor does not match the actual angle.

**Figure 11:**
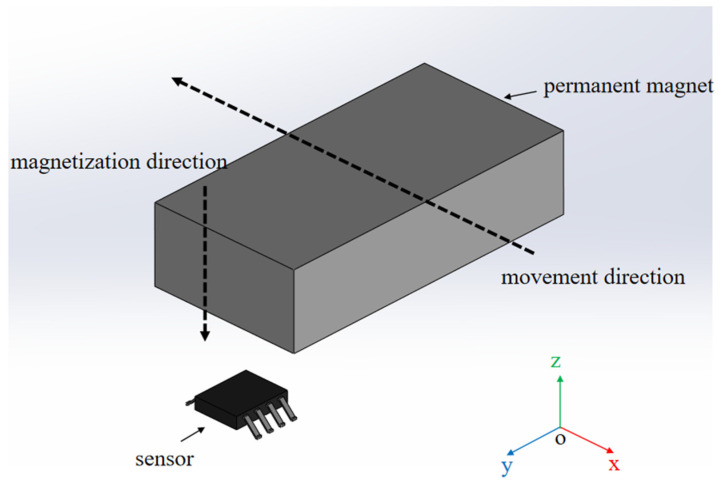
Schematic diagram of the spatial position of the sensor in relation to the permanent magnet in case 2.

Case 2:

Figure 11 illustrates the spatial location relationship between the permanent magnet and the sensor in case 2. In the XY plane, the upper surface of the sensor is parallel to the lower surface of the permanent magnet.

**Figure 12:**
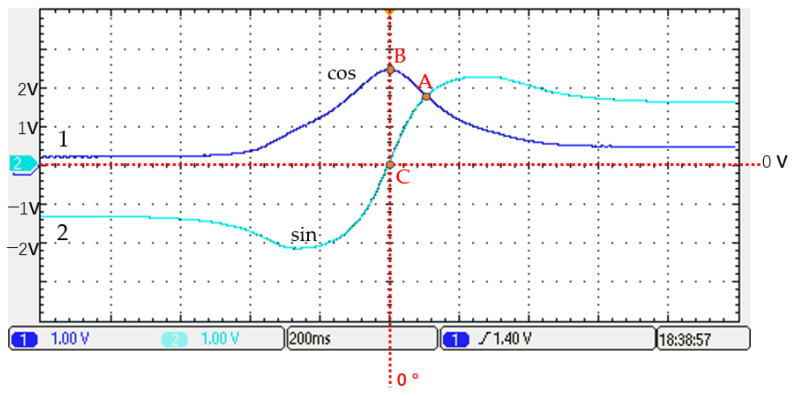
The magnetoresistive sensor output waveform in case 2.

Figure 12 shows the output waveform of the magnetoresistive sensor during the uniform velocity motion of the permanent magnet parallel to the magnetoresistive sensor along a straight line in case 2. Curve no. 2 in the figure shows that the output at point A corresponds to sin (45°), the output at point C corresponds to sin (0°), and the output at point B corresponds to cos (0°).

For the above two mounting methods, the first mounting method of the permanent magnet does not match the actual angular position of the permanent magnet and the sensor and the output angle of the sensor. Therefore, it is not chosen. In contrast, for the second installation method, the permanent magnet matches the actual angular position of the sensor and the output angle of the sensor, so the second one is the preferred choice.

**Figure 13:**
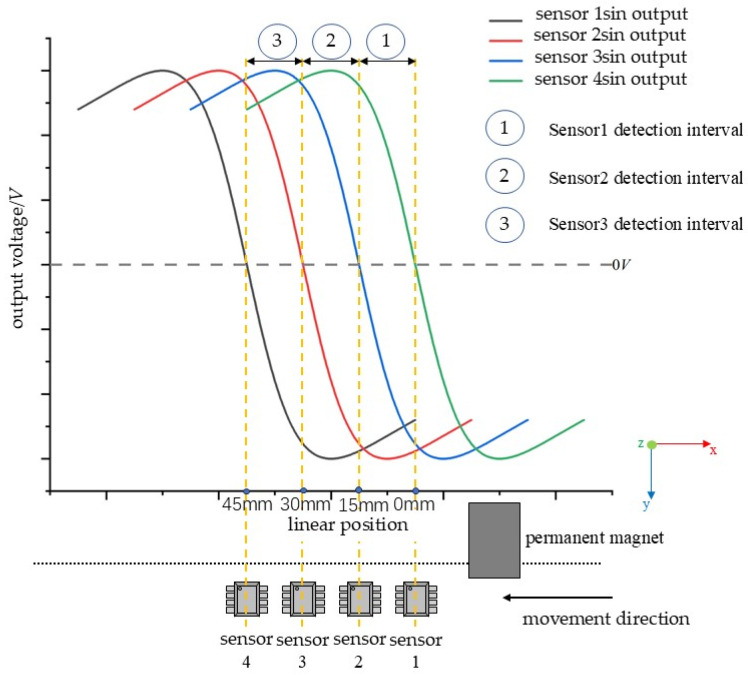
Four consecutive TLE5501 sensor sin output waveforms.

For situations in which there is a piece of permanent magnet in the effective detection range of the TLE5501 magnetoresistive sensor, parallel to the sensor array along the straight line and with movement of a uniform speed, the sin output image of four consecutive TLE5501 sensors is shown in Figure 13.

This system determines the detection interval based on the positive or negative output signal of each sensor. Table 2 shows the output polarity of each sensor in different detection intervals corresponding to Figure 13.

From Table 2, it can be seen that when the permanent magnet is within the detection range of sensor 1, the sin output of sensor 1 is positive, while the sin output of sensor 2 is negative. The sin outputs of sensor 1 and sensor 2 have opposite polarities. The sin outputs of sensor 2 and sensor 3 have the same negative polarity, and the sin outputs of sensor 3 and sensor 4 also have the same negative polarity. Similar situations exist for other detection intervals. By analyzing the sin outputs of adjacent sensors, it can be determined which specific detection interval the permanent magnet is currently located in.

### 2.5. Effects of Different Y and Z Distances on Output Waveforms

**Figure 14:**
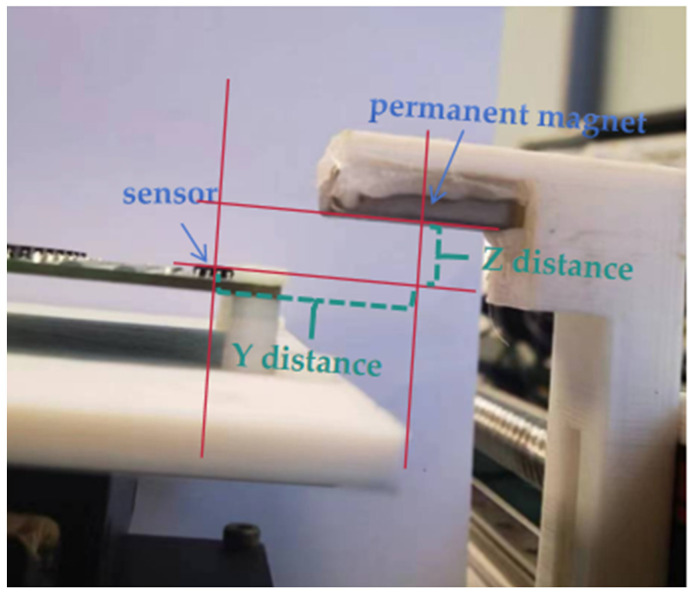
Schematic diagram of the definitions of the distances Y and Z.

The distance from the center point of the sensor to the center point of the permanent magnet along the *y*-axis in the XY-plane is defined as the Y-distance, and the distance from the upper surface of the sensor to the lower surface of the permanent magnet along the *z*-axis in the XZ-plane is defined as the Z-distance, as shown in Figure 14, below.

After determining the relationship between the permanent magnet and the spatial position, it is necessary to adjust the Y and Z distances to determine the optimal measurement distance. During actual measurement, the Y and Z distances cannot be too large; it must be ensured that the sensor is in a saturated state within the measurement range.

**Figure 15:**
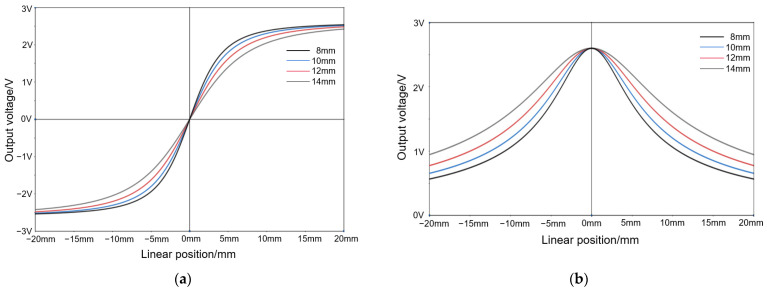
Output waveforms of the TLE5501 corresponding to different Y distances: (**a**) Output waveforms of sin. (**b**) Output waveforms of cos.

When the Z distance is 6 mm, the output waveform of the TLE5501 is measured at Y distances of 8 mm, 10 mm, 12 mm, and 14 mm, respectively. The sin outputs are shown in Figure 15a, and the cos outputs are shown in Figure 15b.

From Figure 15, it can be seen that when other conditions are the same, within a certain detection range, the larger the Y distance, the wider the corresponding straight line detection interval in the same angular range.

**Figure 16:**
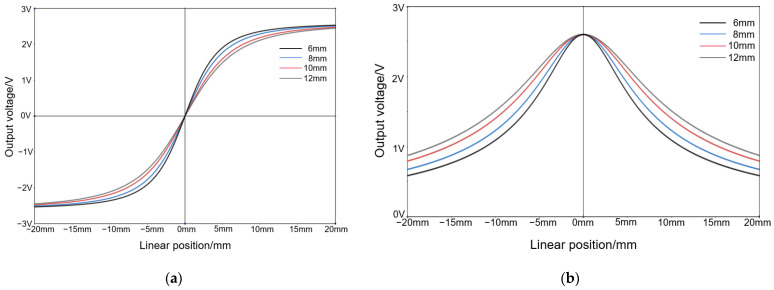
Output waveforms of TLE5501 corresponding to different Z distances: (**a**) Output waveforms of sin. (**b**) Output waveforms of cos.

Under the condition that the Y distance is 15 mm, the TLE5501 outputs certain waveforms when the Z distances are 6 mm, 8 mm, 10 mm, and 12 mm, respectively; the sin outputs are shown in Figure 16a and the cos outputs are shown in Figure 16b:

From Figure 16, it can be seen that in this variation, it is not obvious that the larger the Z distance is, the wider the corresponding linear detection interval is within the same condition of angular range. This condition indicates that within a certain range, the Z distance has almost no effect on the width of the linear detection interval.

### 2.6. Sensor Array Program Principles

**Figure 17:**
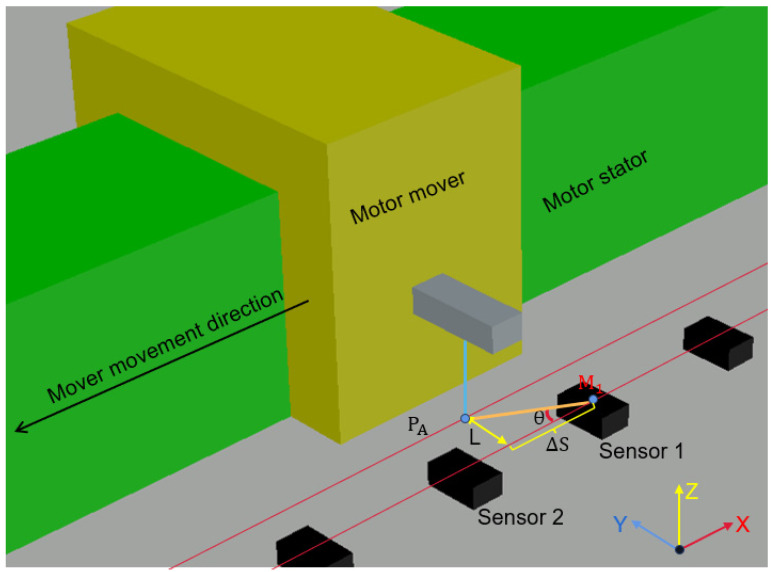
Schematic diagram of the TLE5501 detecting linear position.

The array detection scheme is constructed by splicing multiple single-detection intervals. The displacement detection method for a single sensor interval is shown in Figure 17.

The two bridge outputs of the magnetoresistive sensor TLE5501 are $V_{A}$ and $V_{B}$, which can be converted to an angle θ by arc tangent subdivision:

(11) $$\theta =\arctan(\frac{V_{A}}{V_{B}})$$

The relative position ΔS of the permanent magnet within a sensor detection interval can be calculated by finding the tangent from Equation (12) using simple trigonometric functions, as shown in Figure 17:

(12) ΔS=L×tan(θ)

In the formula, L is the vertical distance from the center point of the sensor to the center point of the permanent magnet.

If the horizontal linear position of sensor 1 is $M_{1}$, the linear position $P_{A}$ of the permanent magnet in a single interval in Figure 17 is

(13) $$P_{A}=M_{1}+\Delta S$$

According to Figure 1, the single-detection interval can be expanded to array detection. Firstly, the absolute positions $M_{1}$, $M_{2}$, …, $M_{i}$ of the center points of each sensor are calibrated by standard length intervals and stored in ROM. After the displacement detection, when the mover is located in the detection range of sensor i, the absolute position *M_i_* of sensor i is read first, and then the angular relationship between the permanent magnet and sensor i is calculated based on the outputs $V_{A}$, $V_{B}$ of sensor i by inverse tangent subdivision. The relative position ΔS of the permanent magnet located in the detection range of the sensor i is calculated by Formula (14), and the linear position $P_{A}$ of the permanent magnet can be obtained by adding the relative position and the absolute position:

(14) $$P_{A}=M_{i}+\Delta S$$

Through the integration of absolute and relative positions, the absolute position of the sensor is reliably recalculated to ensure that the errors of each sensor detection range do not accumulate and overlap with calculations for other sensor detection ranges.

The permanent magnet used as mover in the displacement detection has high remanence, high coercivity, high maximum magnetic energy product, and good material stability. References [36,37] describe the method of calculating magnetic-field strength. The parameters of the neodymium–iron–boron permanent magnet (Zhejiang Sheensen Magnetics Technology Co., Ltd., Zhejiang, China) selected in this article are shown in Table 3.

The normal operating range of the TLE5501 is 20 to 100 (mT). This study collected data on the magnetic-field strength while the mover was in motion within a range of −15 mm to +15 mm, specifically, for the conditions when Y = 8, 10, 12, and 14, and Z = 6, 8, 10, and 12, as shown in the Table 4.

According to the actual data, to ensure that the sensor operates normally, it is essential to maintain the magnetic-field strength within the range of 20 mT to 100 mT in its corresponding detection zone. Given the limited magnetic-field strength of the permanent magnet, both Y and Z should not be too large. A Y position of 10 to 12 mm and a Z position of 8 to 10 mm are considered suitable spatial positions for the sensor relative to the permanent magnet.

## 3. Signal Processing and Acquisition

### 3.1. Signal Amplification Processing Circuit

The TLE5501 magnetic resistance chip outputs two kinds of differential signals. In this scheme, a dual operational amplifier chip LM358 is used to construct two differential amplification circuits. The differential signals output from the two bridges are amplified to an amplitude suitable for A/D acquisition through differential operation and amplification. The filter circuits are added to the power input and signal output in order to reduce signal interference.

### 3.2. AD7606 Analog-To-Digital Converter

The AD7606 analog-to-digital acquisition module is used for multi-channel synchronous A/D acquisition of amplified output signals. The AD7606 analog-to-digital conversion chip can simultaneously perform A/D acquisition on eight channels of signals, so one AD7606 chip can be combined with four TLE5501 sensors.

### 3.3. The FPGA and the STM32 Controller

The FPGA module is used to drive the AD7606 module; it receives the digital quantities obtained from the A/D conversion of the AD7606 module, filters out the sensor outputs of the detection interval where the actuator is currently located by logical judgment, and transmits the digital quantities of the sensor outputs as well as the numbers of the corresponding sensors to the STM32 via FSMC for the subsequent computational processing [38,39].

The STM32 reads the absolute position of the sensor stored in ROM based on the received sensor number, performs an arc tangent operation on the received sensor output, calculates the relative displacement of the actuator in the sensor detection range, and adds the two to obtain the position of the mover of the linear motor. The calculated position of the mover of the linear motor is transmitted to the host computer control system via serial communication [40,41,42].

## 4. System Detection Test

### 4.1. Test Subjects

To verify the feasibility of the non-contact linear motor position detection scheme based on a magnetoresistive sensor array, a linear screw module (Guangzhou CNC Equipment Co., Ltd., Guangzhou, China) was used to simulate the linear motion of the rotor. The screw slide table was installed with the mover, which was made by a 3D printer and embedded with permanent magnets. The screw module was driven and controlled by a stepper motor, with a linear positioning accuracy of 0.05 mm. A magnetic scale model MSR5000(HEIDENHAIN Corporation, Degenfeld, Germany) was selected as the position detection comparison, with an accuracy of ±5 μm. The magnetic grid ruler reading head was installed on the slide table and moved simultaneously with the mover.

**Figure 18:**
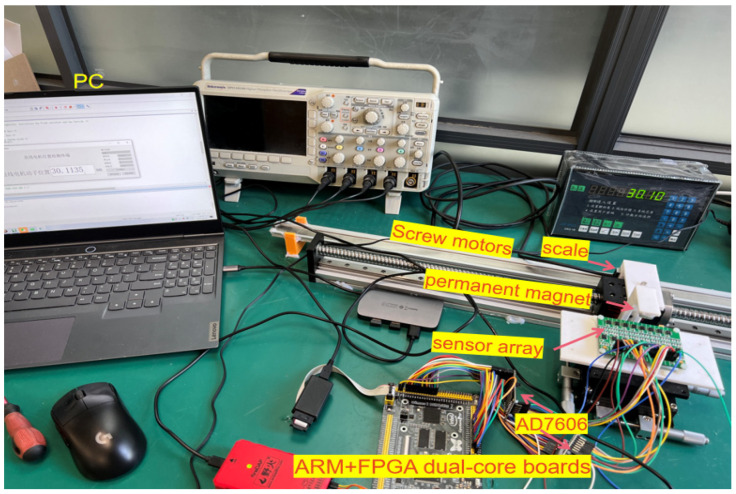
Physical diagram of the test system.

In this experiment, the position detection circuit board was constructed, and eight TLE5501 magnetoresistive sensors were used as a module, with 15 mm spacing for each sensor, and a total of 16 channel output signals. The sensor analogue signals were collected with two AD7606 chips, and the STM32 + FPGA dual core board was used to process the signals and upload the data to the PC to display the current position information. The sensor spacing can be adjusted according to specific requirements, but must ensure that the sensors are kept in a magnetic saturation state within the measurement distance. The physical diagram depicting this arrangement is shown as Figure 18.

In practical applications, the core board of FPGA with multiple IO ports can be used to collect data from various sensor chips simultaneously. Based on the required detection length, it is necessary to deploy a sufficient number of sensor chips and FPGA core boards. When the mover of the linear motor arrives at the interval, the sin and cos signals of the interval sensor are collected and processed by more than one FPGA core board and transmitted to the stm32 for accurate displacement calculation.

### 4.2. Sensor Signal Normalization

**Figure 19:**
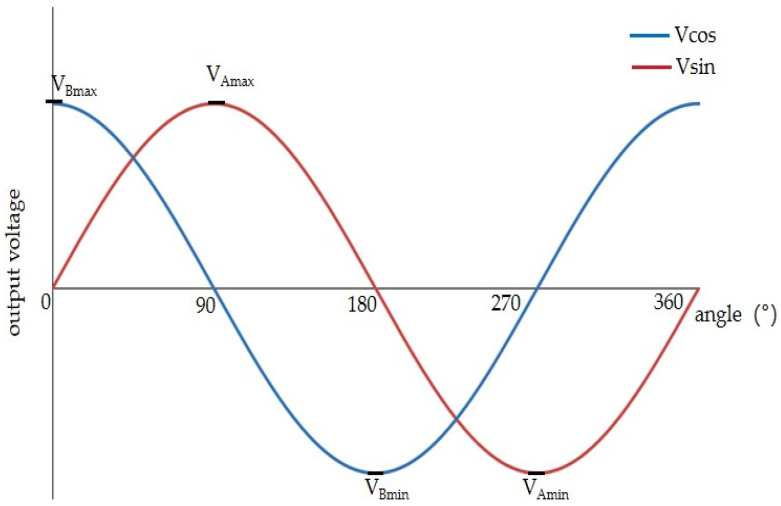
Actual sensor output voltage waveform.

The outputs of each sensor may not be identical, due to the manufacturing process of the chip. Therefore, in order to achieve highly accurate position detection, the two outputs of the TLE5501 magnetoresistive sensor must be normalized. Using a magnet placed directly above the sensor and rotated one period, the trigonometric values of the outputs were obtained as shown in Figure 19; these are in good agreement with Equations (7) and (8).

As for the sin signal, its maximum value is *V_Amax_* and minimum value is *V_Amin_*; the DC bias of the bridge output *V_Aoff_* and peak–peak value *V_Apeak-peak_* are given by

(15) $$V_{Aoff}=\frac{V_{A\max}+V_{A\min}}{2}$$

(16) $$V_{\begin{array}{c} A\mathrm{peak}-peak \end{array}}=V_{\begin{array}{c} A\max \end{array}}-V_{\begin{array}{c} A\min \end{array}}$$

Similarly, the DC bias *V_Boff_* and peak-to-peak value *V_Bpeak-peak_* of the cos signal are

(17) $$V_{Boff}=\frac{V_{B\max}+V_{B\min}}{2}$$

(18) $$V_{\begin{array}{c} \mathrm{Bpeak}-peak \end{array}}=V_{\begin{array}{c} B\max \end{array}}-V_{\begin{array}{c} B\min \end{array}}$$

In the actual measurement, if the peak-to-peak value of the output of the B-bridge is selected as the reference, the output of the A-bridge is defined to be $v_{a}$ and the output of the B-bridge is defined to be $v_{b}$ before normalization. Therefore, the output of the A-bridge is defined as $v_{A}$ and the output of the B-bridge is defined to be $v_{B}$ after normalization, as follows:

(19) $$v_{A}=\left(v_{a}-V_{Aoff}\right)\times \frac{V_{Bpeak-peak}}{V_{Apeak-peak}}$$

(20) $$v_{B}=v_{b}-V_{Boff}$$

The above normalization method solves the problem of unequal DC bias and the unequal magnitudes of the two outputs of the TLE5501 magnetoresistive sensor. By performing the above operation for each sensor, the normalization of the two outputs of each sensor can be realized, which can improve the accuracy of the position detection of the magnetoresistive sensor array.

**Figure 20:**
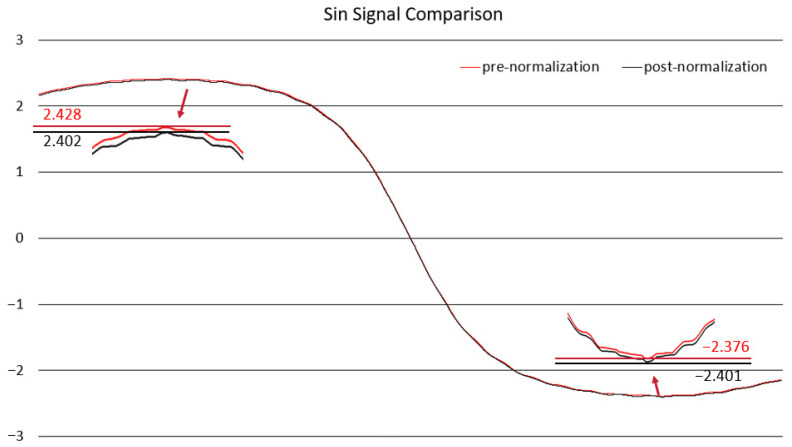
Comparison of sin signal normalization.

**Figure 21:**
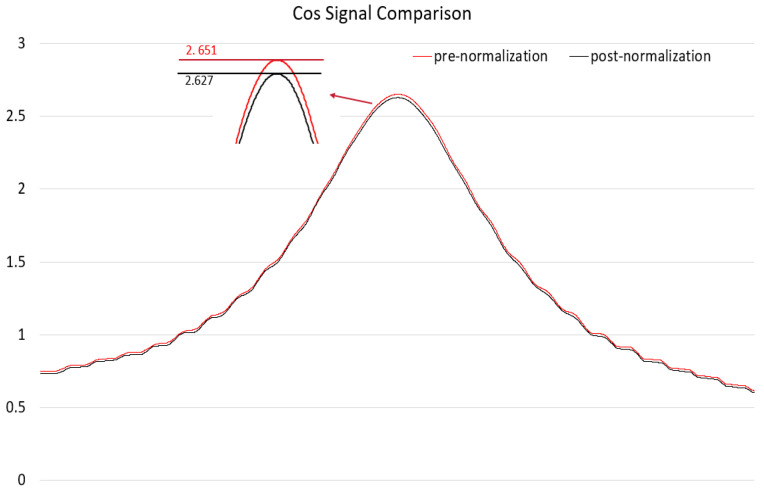
Comparison of cos signal normalization.

In this study, the normalization of the sensor was performed using data from a permanent magnet rotating above the sensor for one complete cycle. Subsequently, the normalized results were validated in the linear displacement detection experiments, as shown in Figure 20 and Figure 21.

According to the experimental data, the normalization of the signals reduced the offset and errors.

### 4.3. Displacement Detection Error Test

The reciprocation error is shown in Figure 20.

**Figure 22:**
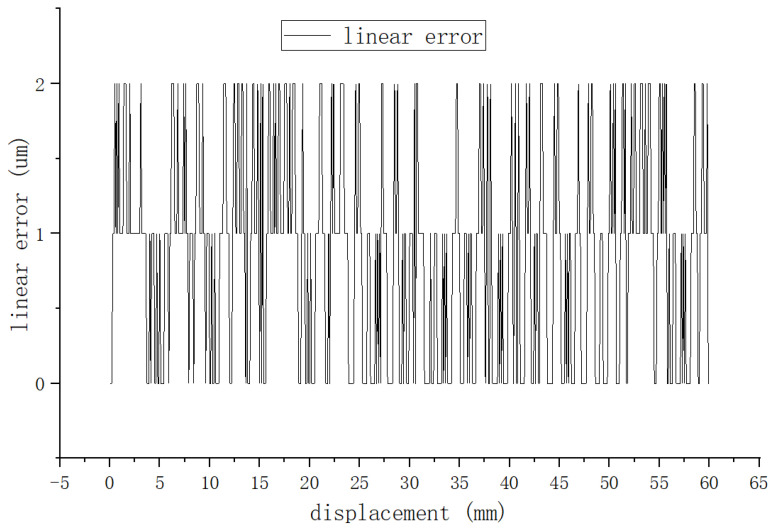
Repeatability detection error curve.

Firstly, we evaluated the stability and repeatability of the system by monitoring for any errors or fluctuations in the position information output by the system as the linear motor was repeatedly moved to the same location. After testing, it was found that the fluctuation was basically maintained in the range of 0~3 μm. The source of these fluctuations is related to the circuit noise interference and changes in the temperature environment. However, the magnitude of these fluctuations does not impact the detection accuracy. The reciprocation error is shown in Figure 22.

During the experiment, the permanent magnet was caused to move from the starting point of the sensor array. Then, a spiral slider drove the permanent magnet linearly along the *x*-axis with a displacement of 0.1 mm per time cycle. At the same time, the PC host computer received and recorded the measured position data and compared the readings with the actual displacement length.

**Figure 23:**
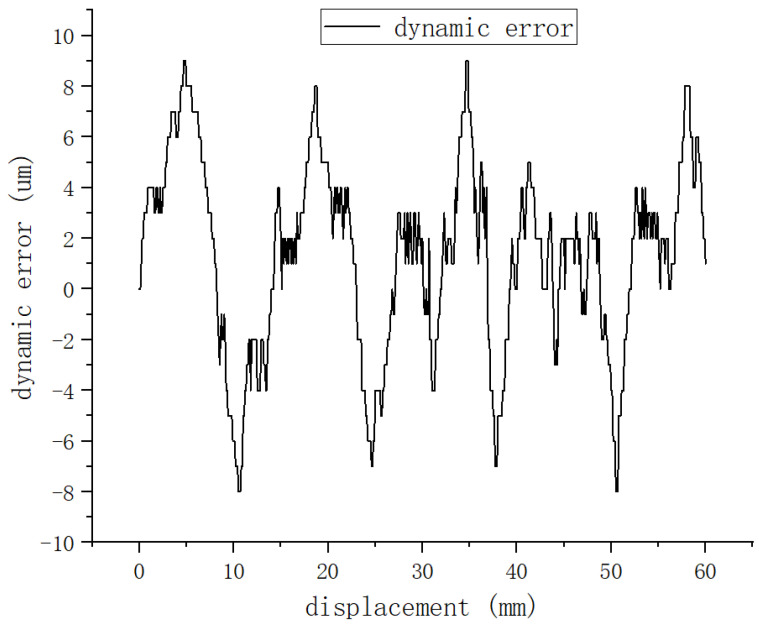
Dynamic error test result graph.

The discrepancy between the measured position and the magnetic encoder’s reading was defined as the dynamic error. Figure 23 shows a graph of the dynamic error test results. Because the spacing of each sensor was recalibrated, only the data from four measurement intervals were selected to illustrate the error range. As can be seen from the graph, the maximum error is within ±9 μm, which meets the accuracy requirements of most industrial applications.

The accuracy is comparable to that of the most widely used magnetic scale. Both can meet most industrial needs. However, this study can fulfill the need for position detection of dynamic magnetic linear motors, a need which cannot be met by magnetic scales under such working conditions.

## 5. Conclusions

This research utilized the characteristics of magnetoresistive sensors to design a mover displacement detection system for a permanent magnet linear motor with a moving-magnet configuration. This paper explains the principle and detection scheme of the magnetic sensor array used for displacement detection. By constructing a dual CPU controller detection circuit, using the Arctan subdivision algorithm, and then normalizing the sensor signal, the absolute position of the mover can be accurately obtained. The magnetoresistive sensor array has the advantages of small physical space, low cost, and easy installation, along with having no need to drag cables; this can help to realize a modular installation and allow the device to effectively detect the absolute position of the mover. This approach provides a feasible method for the position-based closed-loop servo control of moving-magnet permanent magnet linear motors [43,44,45,46].

## Figures and Tables

**Table 1:** The comparison of magnetic sensors.

	TMR	AMR	GMR	GMI
Angle range (deg)	0~360	−90~+90	0~360	0~360
Energy consumption (mA)	1.67	10	10.5	1
Working temperature (°C)	−40~125	−40~125	−40~125	−20~85
Cost	Low	Low	High	Low
Voltage sensitivity (mV/V)	320	24	90	240
Magnetic-field range (mT)	100	20	50	3

**Table 2:** The output of each sensor.

	Sensor 1 Detection Interval	Sensor 2 Detection Interval	Sensor 3 Detection Interval
Sensor 1sin output	Positive	Positive	Positive
Sensor 2sin output	Negative	Positive	Positive
Sensor 3sin output	Negative	Negative	Positive
Sensor 4sin output	Negative	Negative	Negative

**Table 3:** Parameters of NdFeB permanent magnets.

Magnet Name	Parameters	Numerical Value
NdFeB permanent magnets	Residual magnetism	14.2–14.8 KGs
	Coercive force	≥835 KA/m
	Intrinsic coercive force	≥876 KA/m
	Maximum magnetic energy product	390–422 KJ/m^3^
	Magnetization direction	Thickness direction
	Length × Width × Height	25.3 × 12.7 × 6.35 mm^3^

**Table 4:** The magnetic field strength at the location of the sensor when the sensor and the permanent magnet are positioned in different spatial locations.

	Z = 12 (mm)	Z = 10 (mm)	Z = 8 (mm)	Z = 6 (mm)
Y = 14 (mm)	14.8~28.5 (mT)	17.8~33.4 (mT)	19.1~37.2 (mT)	20.8~44.2 (mT)
Y = 12 (mm)	17.2~32.3 (mT)	20.4~44.4 (mT)	24.2~56.4 (mT)	26.4~71.5 (mT)
Y = 10 (mm)	19.6~41.2 (mT)	24.3~54.2 (mT)	27.1~80.6 (mT)	28.8~96.5 (mT)
Y = 8 (mm)	25.6~53.4 (mT)	27.2~76.3 (mT)	32.4~96.4 (mT)	37.5~130.6 (mT)

## Data Availability

Data are contained within the article.
